# Challenges to conducting research on oral health with older adults living in long-term care facilities

**DOI:** 10.1186/s12903-024-04204-x

**Published:** 2024-04-05

**Authors:** Thayse Mayra Chaves Ramos, Álvaro Augusto da Silva Alves, Thais Andrade Apolinário, Flávia Fonseca de Toledo, Viviane Elisângela Gomes, Kevan Guilherme Nóbrega Barbosa, Aline Araújo Sampaio, Raquel Conceição Ferreira

**Affiliations:** 1 Department of Community and Preventive Dentistry, School of Dentistry, Universidade Federal de Minas Gerais, Belo Horizonte, Brazil; 2 Department of Clinical, Pathology and Surgical Dentistry, School of Dentistry, Universidade Federal de Minas Gerais, Belo Horizonte, Brazil

**Keywords:** Oral health, Data collection, Aged, Nursing homes

## Abstract

**Background:**

The challenges to conducting oral health studies involving older people in long-term care facilities (LTCFs) must be debated.

**Objective:**

This study aimed to investigate researchers’ perceptions and experiences while conducting an epidemiological survey on oral health among older individuals residing in LTCFs.

**Methods:**

A qualitative study was conducted involving six researchers who utilized field diaries to record their impressions during data collection through interviews (older individuals (or their proxies), caregivers, and LTCF coordinators) and oral examinations of the older people participants. Additionally, researchers responded to open-ended questions about their experiences. The collected material was subjected to content analysis by two researchers.

**Results:**

The themes that emerged from the analysis were institutional context, aspects affecting the operationalization of the study, and data collection oriented by the clinical-functional profile of the older people. According to the researchers’ perceptions, LTCF coordinators demonstrated concern for the study’s benefits for older adults and the preservation of institutional routines during the research process. Caregivers emerged as vital sources of information, guiding researchers in navigating the challenges posed by the physical and mental complexities of the older people participants, necessitating empathy, sensitivity, and attentive listening from the researchers. The organization of materials and a streamlined data collection process proved essential for optimizing time efficiency and reducing stress for participants and researchers.

**Conclusion:**

The researchers recognized the important role played by LTCF coordinators and formal caregivers, underscoring the significance of empathetic methodologies and streamlined data collection processes in mitigating the challenges inherent to research conducted within LTCFs.

## Background

Population aging is a global phenomenon presenting significant challenges to healthcare systems worldwide due to the burden of chronic age-related conditions [1]. The aging process often leads to frailty and increased functional dependence, resulting in a notable rise in institutionalization [2]. In Brazil, as observed in other countries like the United States, France, and China, the age pyramid has undergone an inversion, contributing to a higher prevalence of chronic diseases and functional dependence among older individuals. Consequently, the population residing in long-term care facilities (LTCFs) has grown substantially [3–5].

Older adults living in LTCFs generally depend more on daily activities than their non-institutionalized counterparts, and oral health is a prominent concern [6, 7]. Institutionalization can negatively impact older individuals eating habits, cognition, and overall functioning, resulting in deteriorating health conditions [6, 8]. Furthermore, significant barriers to oral health care exist within nursing home settings [9, 10]. The oral health of this group is characterized by severe tooth loss, oral diseases, and biofilm accumulation [11–13]. These conditions have been associated with adverse outcomes in terms of general health, quality of life, and mortality [14]. In Brazil, the operations of LTCFs are regulated by the National Vigilance Agency. However, despite the recognized need to improve oral health care provision in these institutions, the regulations do not explicitly address oral health. Therefore, research in LTCFs is imperative to generate robust scientific evidence concerning the oral health needs of older individuals. Such evidence is essential for enhancing standards of care and making informed decisions that prioritize the overall health and well-being of older populations. A recent review analyzing barriers to translating research into oral healthcare policy and practice for older adults stressed the need for increased efforts to undertake research involving older adults, including frail older adults living in residential care, to develop an evidence-informed paradigm for oral health care and expand policies and care practices for this age group [15].

However, conducting studies involving older people in LTCFs poses numerous challenges, demanding meticulous planning, considerable time, and ample resources to overcome these obstacles [16]. Nevertheless, there is a lack of research discussing these challenges [17–19], particularly strategies to include older individuals with dementia in studies [20, 21]. Although health research may share similar challenges, none of these studies have discussed research experiences, including oral health assessment. The previously reported challenges were related to obtaining consent, conducting interviews, engaging caregivers and family members, maintaining privacy, addressing participant attrition, obtaining sufficient sample sizes, accounting for intra-institution cluster effects, dealing with incomplete data, and navigating rigid LTCF practices and routines [16–21]. For older individuals with dementia, researchers emphasize the need for inclusive strategies, considering their communication difficulties, memory loss, diminished autonomy in decision-making, and emotional disposition [20]. The only identified systematic review on methods for involving older people in health-related studies highlights the viability of studies involving older adults, emphasizing the importance of clear communication, building good relationships, and employing flexible approaches [22].

This study aimed to investigate researchers’ perceptions and experiences while conducting an epidemiological survey of oral health among older individuals residing in LTCFs. The findings of this study can provide a valuable understanding of the challenges faced during the study and identify effective strategies to improve the quality and efficiency of future research in this context. Furthermore, understanding researchers’ perspectives makes it possible to develop specific recommendations to enhance research methods for this vulnerable population. By addressing these challenges and designing effective strategies, this research can improve the quality of studies focusing on older populations living in LTCFs and promote evidence-informed oral healthcare policies and practices for this age group.

## Methods

This study employed a qualitative method with a phenomenological approach to explore the experiences of researchers during data collection with older individuals residing in LTCFs and their perceptions of the execution of this work. The phenomenological approach, centered on language, seeks to capture the essence of the lived experience and the emergent meanings from that experience. Previous knowledge of the phenomenon is disregarded to explore how the subjects experience events [23, 24]. Field diaries and an online form with open-ended questions were used to explore the researchers’ experiences.

### Context of study

**Fig. 1:**
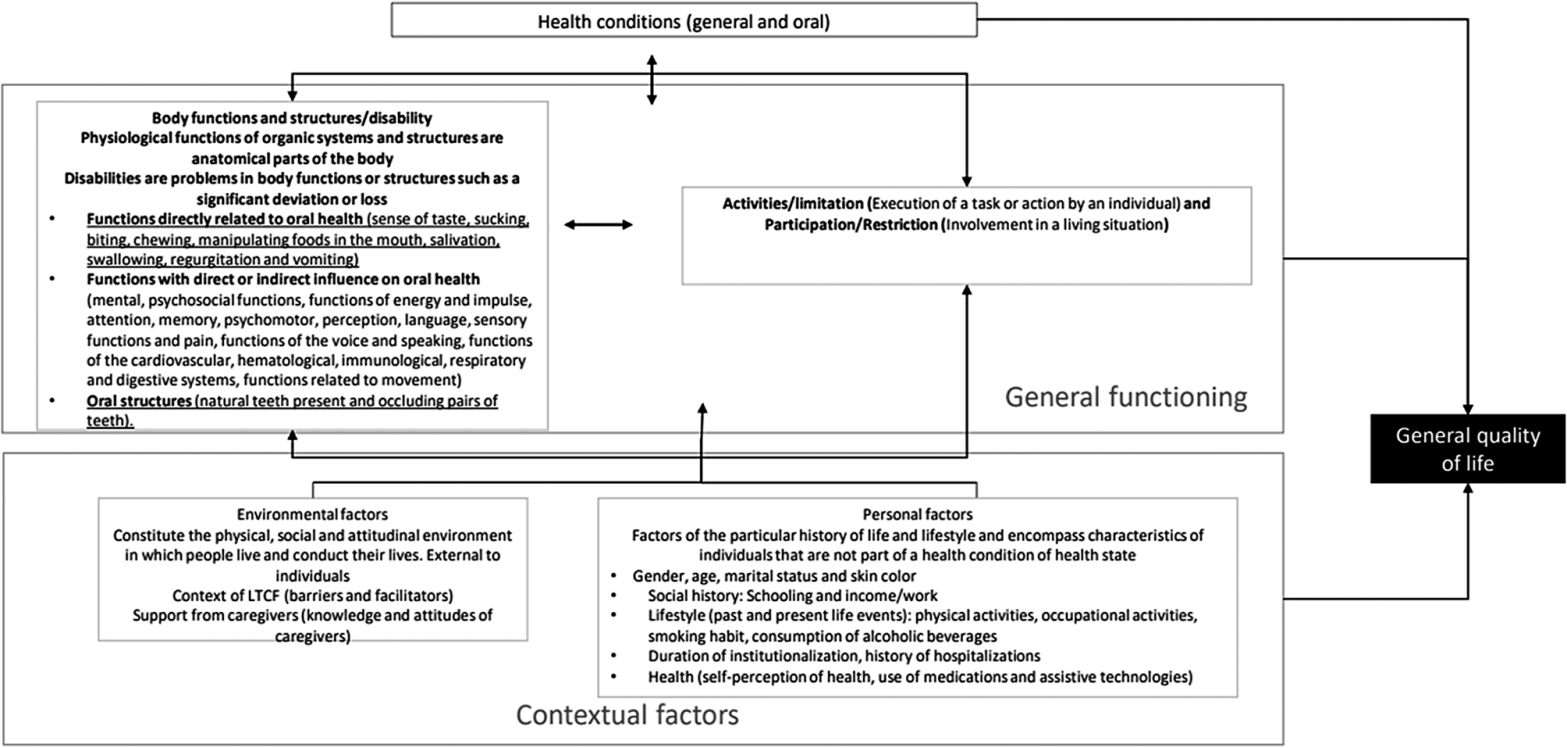
Model adapted from International Classification of Functioning, Disability and Health

The research was conducted at philanthropic LTCFs in Belo Horizonte, Brazil, during a cross-sectional study between August 2022 and March 2023. In 2022, there were 28 philanthropic LTCFs in the city. The sample planning aimed to include all older individuals residing in these facilities, irrespective of their cognitive status. The study participants were coordinators of the LTCFs, formal caregivers of older people, and individuals aged 60 years or older residing in these facilities. The formal caregivers of the older adults were remunerated professionals with employment ties in the LTCFs, having received specific training as elderly caregivers or being nursing technicians. During data collection, they assisted and cared for the older adults. Epidemiological data were collected through interviews with the coordinators, formal caregivers, and older individuals or their proxies (caregivers). The collected variables followed the model of the International Classification of Functioning, Disability, and Health (Fig. 1), which included anthropometric measures and physical and oral examinations conducted at the LTCFs.

The six researchers involved in the study had undergone prior training for conducting interviews, and four of them received calibration exercises for the oral examinations. All six researchers participated in the data collection process for the epidemiological research. These researchers consisted of both undergraduate dental students and master’s degree graduate students, who formed pairs to serve as interviewers, examiners, and/or assistants (annotators).

A pilot study was carried out at one of the LTCFs participating in the research to ensure the smooth execution of the study. This pilot study allowed for testing the digital data recording tools and refining the sequence and dynamics for conducting interviews and examinations. The pilot study served as a preparatory phase, ensuring the research procedures were well-coordinated and optimized before the main data collection phase.

### Procedure and participants

The research utilized a field diary as the primary method to record informal conversations, observations of the behavior of older people and formal caregivers during data collection, reflections on the examination process and methods employed, as well as the researchers’ impressions regarding the data collection process within the LTCF setting [25, 26]. Researchers independently and freely made digital-format entries in their respective field diaries.

All six researchers independently and freely made digital-format entries in their respective field diaries. Criterion sampling was the method utilized for selecting this sample, which encompassed all researchers who have shared an experience, yet exhibit variations in characteristics and individual experiences [27]. In addition to the field diary, an online form with open-ended questions was employed to collect individual feedback from each researcher about their feelings and experiences as a researcher during the fieldwork. The form included the following guiding questions: (1) How was your experience collecting data at the LTCFs, considering the older people, caregivers, and LTCF context? (2) What was the most striking aspect during the days you collected data at the LTCFs? (3) If you were to advise a researcher about beginning data collection at a long-term care facility through interviews with older people, what observations would you share to ensure their success? (4) what is the main aspect that should be considered for satisfactory data collection with older people similar to those encountered at the LTCFs? The responses to these questions contributed to the researchers’ reflections and perspectives. They were considered part of the corpus of analysis for the study.

### Data analysis

The contents of the field diaries and open-ended questions were independently submitted to exhaustive readings by two researchers with experience in qualitative studies for a more in-depth capturing of the information. Subsequently, the data underwent content analysis, following the approach proposed by Graneheim and Lundman [28]. The researchers identified units of meaning within the records and extracted the essence of each unit, resulting in the creation of condensed units of meaning. Through this process, categories and themes that emerged from the analyzed content were identified. Reliability was ensured through continual discussion of the data with the team. Consensus meetings were held to ensure agreement on the themes that emerged. In the final analysis, codes such as R1, R2, and so forth were used to represent each of the interviewees, allowing for a systematic and organized representation of the participants’ contributions.

### Ethical aspects

This study received approval from the Human Research Ethics Committee of the Universidade Federal de Minas Gerais. The participants signed a statement of informed consent.

## Results and discussion

The data collection for the epidemiological study on oral health assessment in 14 LTCFs, 311 older people, and 164 formal caregivers involved six researchers. They recorded their observations in field diaries and responded to open-ended questions. Through content analysis of the field diaries and open-ended questions, three main themes emerged: (1) institutional context, (2) aspects affecting the operationalization of the study, and (3) data collection oriented by the clinical-functional profile of the older people. The categories under each theme are presented in Table 1.

**Table 1:** Themes and categories summarizing the perceptions of the researchers while collecting epidemiologic data on the oral health of older people living in long-term care facilities (LTCF)

Categories	Themes
Social role played by LTCFs	Institutional context
Ambience of LTCFs	
Clinical-functional profile of older people living in LTCFs	
Oral health of older people living in LTCFs	
Access to oral care by older people living in LTCFs	
Acceptance of LTCFs to participate	Aspects affecting the operationalization of the study
The impact of institutional routines on the research process	
Data collection location and methods	
Approaching the residents	Data collection oriented by the clinical-functional profile of older people
Respect for the autonomy of the residents	
Communication with the residents	
Caregiver’s knowledge	

### Institutional context

Results regarding the institutional context are presented in Table 2, showcasing the units of meaning that illustrate the categories within this theme. The researchers recognized the crucial *social role played by LTCFs* in reintegrating older people, particularly those who have experienced neglect or loneliness, perceiving these institutions as mandated by Brazilian legislation to care for and support older individuals [29]. Regarding the *ambience of LTCFs*, the study revealed a wide variation in the activities and services offered to residents across different institutions. As stipulated by the Brazilian Resolution, LTCFs should provide a welcoming environment that upholds older people’s human rights and dignity, including aspects such as identity, freedom of beliefs, freedom to come and go, privacy, and respect [30]. The ambience also encompasses fostering family and community involvement in caregiving, the coexistence of residents with different degrees of dependence, supporting residents’ autonomy, promoting leisure opportunities, and preventing violence or discrimination against residents [29]. The Brazilian regulation also standardizes structural aspects of the LTCFs, human resources, health care, nutrition, washing, processing, and storage of clothing, and cleaning facilities [29]. The researchers’ perceptions indicated the importance of establishing systematized assessment processes to reveal the different levels of quality of the LTCFs, indicating the need for policies that favor achieving the principles of ambience and the well-being of the older people who reside in these facilities.

The *profile of the older people living in the LTCFs*, as recorded by the researchers, was characterized by high frequencies of cognitive impairment, clinical-functional frailty, mental and behavioral disorders, and dependence in performing basic and instrumental activities of daily living. These characteristics posed challenges during the data collection process, as many participants exhibited refusal, resistance, and low levels of cooperation with the study due to their health conditions. This clinical-functional profile is similar to that described for older people living in long-term care facilities worldwide [8, 14]. The researchers also observed rapid functional decline among the older people during the data collection period when the same individuals were visited on different occasions from one week to the next. As a cross-sectional study, the researchers sought to conclude data collection in the first and only approach to older people, whenever possible.

Moreover, fluctuating interest in participation necessitated additional attempts to secure their involvement due to emotional and health-related fluctuations. A previous study involving older people with dementia found that verbal communication varied between weeks, from one day to another, and even within the same day [20]. Another challenge was dealing with the losses of individuals. Data collection began with mapping all residents at the institution by consulting the records. When seeking older people for interviews, there were cases of death – either recent or longer ago. In the latter case, it was perceived that the LTCFs did not perform regular updating and the separation of records.

Regarding *oral health*, the researchers identified a precarious situation among older people, with a high frequency of tooth loss, caries, and periodontal disease in the remaining teeth, along with unsatisfactory dental prostheses and accumulation of biofilm and dental calculus. This oral health profile aligns with previous studies in different countries, highlighting the substantial burden of oral diseases among institutionalized older individuals [13, 31, 32]. This researchers perception reflects the oral health profile of older people living in LTCFs in Belo Horizonte for more than a decade and a half [12], demonstrating that this population is a special needs group requiring oral care improvements [11, 31].

The researchers recorded the difficulty of older people accessing routine oral care, as many depend on caregivers who have an excessive workload, have little time available to perform oral hygiene, do not prioritize it, or are unaware of its importance. A daily routine of oral hygiene during bathing was often observed, as reported in a previous study, in which nurses reported that the teeth of the majority of residents were brushed at least once a day [13]. The researchers’ findings underscore the pressing need for improved oral care for this special needs population, emphasizing the importance of incorporating oral hygiene into routine healthcare practices, promoting oral care initiatives, and providing training for caregivers [13, 33].

**Table 2:** Categories and units of meaning extracted from the researchers’ records on the ‘institutional context’ theme

Theme: Institutional context	
Categories	Units of meaning
Social role played by LTCFs	*The institutions play a fundamental role in the reintegration of older people who, in many cases, experienced a situation of negligence and loneliness.* (R1)
Ambience of LTCFs	*Each institution has a schedule and routine, with fun activities, such as singing circles, bingo, reading sessions, and stimulating activities, such as physiotherapy and activities that simulate school classes.* (R3) *The caregivers perform dances, music and provide beauty care for the residents*. (R5) *The LTCF has a large space in which the residents can walk around freely. Others are very small, with no adequate open space that fits all the residents. Some have a leisure area with comfortable armchairs for each resident, a TV area, large rooms with two beds; others had rooms with five beds and no adequate leisure area*. (R6)
Clinical-functional profile of older people living in LTCFs	*The rapid decline in the health status of some residents was also an obstacle in some homes; we arrived on one occasion for data collection and when we returned, we were unable to continue the process with some of them because they had been hospitalized, had died or were bedridden.* (R3)” *Trying to collect data on more than one occasion; on some days, the resident refused or did not demonstrate any interest in answering. It is important to respect this. However, the same person is willing to participate on another occasion.* (R4) *(…) some residents died during the period of the study.* (R5) * Many do not know where they are, what year it is, what day it is. They live in their own world.* (R3) *The issue of dementia combined with the senility process and the fact that the majority of institutionalized older people have grade 2 and 3 dependence and are cognitively compromised, which was evident during the administration of the Mini Mental State Examination and in situations of moods swings and traces of violence that we witnessed.* (R1) *It was not possible to perform the clinical examination, since cognitive capacity was very affected. Some refused to cooperate, did not understand the purpose of the tests, became weary during the steps, deviated the purpose of the visit to talk or were even violent.* (R6)
Oral health of older people living in LTCFs	*We found most residents with needs for complete dentures, with ill-fitting, loose, worn, old dentures without chewing function and dirty. Those with teeth had calculus, active caries and root remnants marked by inflamed periodontal tissues. There were also residents with hyperplastic lesions and fungal infections in the perioral region, angle of the mouth and even in the submandibular region.* (R4) *The quantity of residents that do not have teeth and do not adapt to the prothesis, the lack of hygiene of the prosthesis and teeth, the quantity of plaque and calculus on the teeth is astonishing.* (R1) *We witnessed the breakdown of one woman, who we later found out was schizophrenic. When we talked to her and performed the examination, her behavior and response were extremely calm. She was very responsive and cooperative as well as affectionate. The episode of being out of control was marked by throwing objects, pushing chairs, screaming and agitation. It was apparently for having been left out of a ‘selfie’ that took place among a group of residents.* (R2)
Access to oral care by older people living in LTCFs	*The caregivers do not pay proper attention to the brushing of the prostheses and teeth of the residents – whether due to a lack of time or knowledge.* (R5) *Hygiene is complicated. It is done once a day at bath time. There are many residents and most depend on the assistance of the caregiver, which impedes brushing more times a day.* (R6) *Many residents can perform their own oral hygiene, but the fact that the caregiver does everything, the residents begin losing their autonomy and leave oral hygiene up to the caregiver, who often is unable.* (R3)

### Aspects affecting the operationalization of the study

The researchers encountered various challenges related to the operationalization of the study, particularly in gaining the acceptance and cooperation of LTCFs. Results regarding the operationalization of the study are presented in Table 3. The signing of informed consent by the LTCF coordinators proved to be a complex process, with many expressing resistance and skepticism about the study’s potential impact and benefits for the older residents. Some questioned the importance of a study involving individuals at the end of life. The researchers faced concerns about interrupting institutional routines and potential risks to the residents without any direct return. Similar challenges have been observed in studies conducted in the United Kingdom, where the presence of researchers was perceived as intrusive [17]. In compliance with Brazilian legislation on research involving human beings, participation must be consented to clearly and voluntarily with no financial compensation. As experienced by other researchers, flexibility and creativity were needed to justify the importance of the project to generate evidence that reinforces the importance of oral health for this group. It was also essential to emphasize the low risk associated with the participation of older individuals [17]. Establishing a trusting relationship with the coordinators of the LTCFs proved vital for their willingness to participate in the study. The researchers tried to showcase the study’s potential in generating valuable knowledge, organizing academic extension activities tailored to this specific population, and the potential benefits it could bring to enhance resident care. The presence of researchers might have encountered increased resistance during the pandemic, leading to visit cancellations due to concerns about the higher risk of mortality and morbidity from the coronavirus among older people [34].

The study planning at LTCFs should include the time spent on recruitment and the need for different approaches for contact: repeated telephone calls, personal visits, the presentation of documents/written projects, and the joint determination of a data collection timeframe. Researchers should also be prepared to deal with refusals, as occurred in this study when coordinators vehemently refused to participate, stating that they did not have the authorization or that the LTCF was part of a network that did not permit study participation. This challenge shows that building collaborative relationships with LTCFs is essential to understand research concerns clearly and to plan a project involving vulnerable adults jointly [15]. In contrast, the researchers also recorded situations in which the coordinators were receptive to the study, recognizing that it is important to demonstrate the needs of this population, which could result in programs and policies for older people who reside in LTCFs.

The process of obtaining informed consent from the older residents themselves was also challenging, especially for those with severe cognitive impairment. In such cases, consent was given by caregivers or LTCF coordinators acting as guardians of the older people. The issue of consent by proxy and the ability of the proxy to represent the wishes of cognitively impaired adults has been a subject of debate [15]. Several researchers have highlighted the challenges of obtaining informed consent and respecting the autonomy of individuals with dementia [16–21, 35]. Hubbard and Maas emphasized the importance of continually monitoring the individual’s desire to participate, even when a proxy provides consent through the interpretation of verbal and nonverbal signs. They asserted that consent is an ongoing process rather than an a *priori* one-time event, as Crossan & McColgan (1999) mentioned. We encountered similar situations where residents could not provide direct consent. In such cases, the researchers took great care to explain the study and obtain their assent while interpreting their facial expressions and behavior to respect their autonomy and wishes. Despite these efforts, 15 invited older people chose not to participate.

The *institutional routines* of LTCFs significantly impacted the study execution. Researchers had to consider and respect the schedules and activities of the older residents, leading to adjustments in the data collection timeframe. Additionally, finding suitable times for interviews and examinations was challenging due to the residents’ mobility problems and caregivers’ availability. The researchers had to collaborate with LTCF coordinators to find mutually agreeable time slots while ensuring minimal disruption to the institution and its residents. Finding suitable time slots to conduct interviews was also a significant challenge, as observed in a previous study exploring the perception of dignity among older people residing in LTCFs [19]. The authors of that study emphasized the need to avoid peak activity times, such as meals or regular visits by physicians, and to avoid conducting interviews immediately after an activity, such as lunch, as participants often displayed weariness and lethargy during such periods [19]. To overcome this challenge, the research team collaborated with the LTCF coordinators to agree on appropriate data collection times that did not disrupt the institution’s routines or inconvenience the residents. This required a significant consideration of each location’s availability and the staff’s workload. Other researchers have noted these challenges [17, 19–21, 35], especially considering that caregivers are crucial as proxies for older people. Researchers also had to contend with the unavailability of caregivers to answer questions due to their multifaceted responsibilities in caring for many residents. Introducing the study could thus be an additional burden for them, which many might perceive as unwanted.

In addition to respecting the institutional dynamics, the execution of data collection required careful organization by the researchers regarding the selection of *data collection location and methods*. Many residents faced mobility issues, making moving from one place to another challenging. In some instances, caregivers were unavailable to assist in this task, requiring additional time to reach the most suitable location for the interview or oral examination. Factors such as lighting, privacy, and participant comfort had to be considered during this process. Adapting the data collection process to the specific situation encountered at each LTCF was necessary. Some facilities had designated spaces for the study, while others lacked appropriate areas, leading to examinations being conducted wherever possible, such as in TV armchairs or beds. Previous studies have discussed the need for such adaptations [17, 20]. According to Hall, Longhurst and Higginson, these field situations also posed challenges to maintaining privacy during data collection, which became especially sensitive during oral examinations [19]. The proximity required for oral examinations could generate discomfort, mainly when conducted in the presence of colleagues and staff. Efforts were made to ensure privacy in such situations. Consequently, conducting studies in this context demanded considerable flexibility and reciprocity, considering the limitations and demands of the LTCFs [19]. The researchers were also concerned about biosafety and cross-infection prevention [36], mainly due to the vulnerability of older people to the COVID-19 pandemic. Adhering to strict protocols and protective measures during data collection became essential to safeguard the health of both residents and researchers.

Various measures can be employed to ensure standardization and successful data collection. Providing proper training and ongoing supervision for the researchers is essential. This training should cover all aspects of the data collection process, including interview techniques, oral examination protocols, and ethical considerations. Additionally, it is crucial to ensure that the researchers have access to the minimum necessary resources required for data collection, such as sterilized clinical kits, personal protective equipment, and appropriate data recording tools. A comprehensive manual of norms and standard procedures should be prepared to maintain consistency and adherence to established protocols. This manual should outline step-by-step instructions for each stage of the data collection process, from participant recruitment to data recording and analysis. Regular reference to this manual will help researchers follow standardized procedures and minimize the risk of errors or deviations during the study [37].

The high proportion of older people with cognitive impairment created additional complexities. Variables related to subjective aspects, such as quality of life and self-perception of health, posed challenges since some residents had limited discursive capacity. The researchers utilized validated instruments designed for older adults with adequate cognitive levels but recognized the need for more context-specific tools for individuals with dementia. Challenges to assessing subjective aspects of the lives of older people with dementia have been discussed, considering the lack of validated instruments for this population. There is a debate in the literature on whether data collected from individuals with dementia are reliable due to cognitive impairment [38]. However, more recently, there has been growing recognition that such individuals can express perceptions, needs, and concerns [38, 39], and their subjective experiences should be considered and investigated in studies [20, 39]. Approaches such as structured observation focused on nonverbal communication (facial expressions and body language) and nonstructured observation within the ethnographic tradition have been employed in previous studies to understand the social world of older people [20]. A study assessing quality of life among older people with dementia combined observation with interviews using open-ended questions, and older people were included based on their capacity to communicate verbally in a conversation rather than based on the diagnosis of dementia [20]. The literature describes the need to use multiple (qualitative and quantitative) methods in studies involving individuals with dementia with different levels of verbal communication skills to promote a contextualized, multidimensional assessment [39]. Such approaches should also be considered strategies to understand the quality of life in the context of oral health assessments in future studies and to guide care strategies considering the experiences and wishes of individuals with dementia.

**Table 3:** Categories and units of meaning extracted from the researchers’ records for the ‘operationalization of the study’ theme

Theme: Operationalization of the study	
Categories	Units of meaning
Acceptance of LTCFs to participate	*The caregivers/coordination were quite receptive and understood the importance of gathering data to the quality of life of the older people who reside there. The fact that we could offer more palpable benefits in return, such as a training course for caregivers, was also an important incentive to participation.* (R6) *Contact with some LTCFs and the authorization for use to go to the homes was difficult, since there was a certain resistance justified by renovation work, the incompatibility of schedules and even fear of a lack of return from the study for the institution, as well as a conflict of schedules in one case.* (R4) *Some coordinators took a long time to answer our telephone call. They are always very busy, ask us to call back another time and, when we call, they are no longer available. Others report not having the authority to make decisions and transfer us to another sector of the LTCF, making acceptance difficult and delaying the beginning of the data collection process.* (R1) *Convincing the residents to participate in the study required greater sensitivity to explain, to show the reason for doing the tests, what each part meant in terms of their performance… it ends up diminishing productivity.* (R5)
The impact of institutional routines on the research process	*Being aware of the policies of the institution and that the rules established for interaction with the residents are followed.* (R2) *The schedule of the institutional dynamics; when we would arrive for data collection and after a short while we had to stop because it was time for afternoon coffee, dinner, bath, etc. Some of them limited the days and the quantity of people that could enter the institution.* (R1) *We had few hours to perform our tests, since some institutions limited the visit to only the morning or afternoon and we could not interrupt the activities of the day or compromise the schedules. For instance, we would have to stop in the morning at 11 o’clock, because it was lunchtime and at around 5 pm in the afternoon because it coincided with afternoon coffee and the time for preparation for rest.”* (R3) *These employees (caregivers) are under constant pressure with an excessive workload and older people to take care of. They work in shifts and, to hold interviews with all of them, it is necessary to organized, to go at different periods and days or to leave a questionnaire for them to answer it a little at a time.* (R5) *Most of the coordinators of the LTCFs understand the importance of the study for the older people and for the LTCFs, but become distressed regarding how to receive the researchers without interfering with the routine of the home and therefore end up restricting the days of the week and times for receiving us.* (R3)
Data collection location and method	*Having a specific location to perform the clinical examination facilitates the data collection process. It is easier for a caregiver to bring the resident to a location than for the researcher to take all the material to the resident. Often there is no adequate area to support the instruments and this moving around requires time.* (R2) *The fact that we don’t have a space for collecting data makes the dynamics very complex and slow, because we have to go to the resident, who is often sitting in the TV room and our material is in another place.* (R3) *Collection itself was somewhat perturbed due to not having a fixed location. So, we used a sofa that was near the residents in the TV room and we adjusted to the situation.* (R5) *Following hygiene rules since when performing oral examinations in a long-term care facility, it is important to follow safety and hygiene rules to protect both the participant and researcher. Remembering the use of PPE, keeping the hands sanitized and taking care to avoid cross-contamination among the participants.* (R2) *Good lighting and attention to ergonomics are necessary.* (R3) *Having a clear routine and organization to follow during data collection so that you can collect all the necessary information without losing time or becoming confused.* (R1)” *The team needs to be trained and with a minimum number of members. A lack of researchers has an impact on the daily result of data collection.* (R5)” *The residents were not able to answer many of the questionnaires and a proxy informant was necessary. So, we’re not going to be able to assess many questions that only older people with preserved cognitive could answer, such as questions about quality of life.* (R6)”

### Data collection oriented by the clinical-functional profile of the older people

The researchers revealed that the *clinical-functional profile of older people* requires differentiated approaches for data collection, particularly the use of relational skills, such as empathy, active listening, patience, sensitivity, and flexibility to deal with different behaviors – ranging from cooperative to resistant individuals. Results regarding the data collection oriented by the clinical-functional profile of the older people are presented in Table 4. Cognitive impairment and levels of cooperation were identified as obstacles to the data collection process, with frequent resistance to the study. The progression of cognitive decline leads to a deterioration of cognitive functions and behavior and mood disorders, including depression, irritability, and aggressiveness [40–42].

This profile of the older people also required *communication* strategies on the part of the researchers, who needed to be direct and clear, often involving the participation of the caregivers. The researchers manifested insecurity, feeling unprepared to understand and deal with older people in some situations. Hubbard, Downs, and Tester [20] suggest that researchers dealing with dementia should be trained as skilled verbal and nonverbal communicators, sensitive to how dementia impacts memory, decision-making capacity, and emotions. Developing strategies tailored to each participant’s unique experiences and listening to their voice is essential. Hall, Longhurst, and Higginson [19] add that researchers must be particularly patient, and the extra time and training for this must be built into the research design. Establishing set protocols for handling various responses ensures uniformity and consistency [19]. Researchers also had to contend with parallel conversations,” where older people spoke about other subjects and extended the conversation. This required employing different communication strategies and striking a balance between listening to the older person and returning to the assessment without causing discomfort [16, 20].

Sensory impairments, such as low visual and hearing acuity, also compromise communication. Hearing impairment is common among older people [43], and there are also high proportions of blindness and vision impairment among residents of LTCFs [44, 45]. Interviews involving older people with hearing impairment were found to be draining, as the researchers needed to raise their voices and repeat questions. This limitation can negatively impact the quality of dialogue and create discomfort for the older person [20].

The researchers highlighted the caregivers’ knowledge in guiding the data collection process according to the clinical-functional profile of the participants. Being familiar with older people and their physical and mental status, caregivers served as valuable mediators, offering insights into effective communication and strategies for dealing with each case. Caregivers of older people perform the functions of accompaniment and care, offering emotional support as well as support in their social interactions, assisting and accompanying routines of personal and environmental hygiene, nutrition, preventive health care, the administration of medications and other health procedures, and assisting and accompanying the mobility of older people in activities of education, culture, recreation, and leisure [46]. Moreover, caregivers also acted as a proxy for older adults with cognitive impairment, providing information about health and daily activities. The researchers recognized caregivers as a source of support during data collection, contributing to a more efficient and enjoyable data collection process by helping identify older people and guiding them to data collection locations.

**Table 4:** Categories and units of meaning extracted from the researchers’ records for the ‘data collection oriented by the clinical-functional profile of the older people’ theme

Theme: Data collection oriented by the clinical-functional profile of older people	
Categories	Units of meaning
Approaching the residents	*Approaching the residents with respect and cordiality, always taking into consideration their physical and cognitive limitations.* (R5) *Having patience, being calm and receptive, knowing how to listen, because they like to talk and interact. It is important to allow them to express themselves, but you also need to get back to the study. On the other hand, others do not want to communicate and this must be respected. After all, irritating them has a negative impact on the environment.* (R1)” *It is necessary to be patient during all interactions, regardless of the level of cooperation, since it is the individuality of each person, depending on their physical, cognitive, social and cultural limitations.* (R2) *Flexibility is important, because each one has his or her own needs and limitations.* (R3) *Empathy, care and knowing how to listen, to care, have patience and be practical.* (R1)” *Quickly tiring (the collection time needs to be short): In addition, some residents begin to question the methods of our study; they begin to collaborate and become tired in the process.* (R4)
Respect for the autonomy of the residents	*Older people have the right to decide whether or not they want to participate in the study and their decisions must be respected.* (R5) *Even those without preserved cognition, it is necessary that they want to participate and are cooperative with the process of the study.* (R1)
Communication with the residents	*Communication with some residents is complicated – whether due to speech difficulties or a lack of interest.* (R4) *Understanding that they (older people) have their needs, their conceptions of the world, being from different generations, their expectations with regards to visitors and what they represent.* (R1) *Playing the role of listener for the residents, who, in most cases, want to have parallel conversations.* (R3) *The biggest difficulty was having patience, knowing how to talk, give information/instructions such that the resident is able to perform what was requested.* (R5) *In general, communication is a little complex because some have difficulties expressing themselves or understanding what we are saying. Sometimes we also have difficulty understanding what they say, because there is not much sense, or be able to communicate, talk with them. Some get angry.* (R3) *I often feel unprepared to deal with the residents. A researcher needs to be prepared to understand the different behaviors and reactions, generally of dementia, and to deal with surprises and mood swings.* (R2) *Sometimes we’re are talking, the resident nods his head as if he understands, but he doesn’t. It is necessary to speak close to their ears and repeat the words so that they understand.* (R1) *Difficulty seeing was something that we perceived in a large part of the residents when we asked them to write something or execute some command; many said that they had cataracts, glaucoma and didn’t see well.* (R2)
Caregiver’s knowledge	*They can be a great source of support during the data collection process and can provide valuable information on the behavior of the residents and their daily needs.* (R2)” *I counted on the active help of the caregivers, which made data collection faster and pleasurable.* (R1) * Caregivers can clarify the real needs of the residents. For instance, some questionnaires have items addressing whether the person serves his own food or not. At some institutions, this process is a protocol and meals are served to the residents. However, some have complete autonomy to perform this action alone. So, these points need to be clarified to ensure an accurate assessment of the level of dependence of each one.* (R3)” * We came across new situations. We were alerted by the caregivers about more than one resident with aggressive behavior – from verbal to physical aggression – due to some cognitive impairment, who, in order to be treated, may require arm restraints.* (R2)”

The research techniques proved suitable and valuable for understanding the researchers’ experiences. The records of these experiences revealed various challenges and strategies in conducting studies involving older people residing in LTCFs, considering the diversity of residents’ profiles and the institutional context. Table 5 presents a synthesis of the main challenges and the strategies employed to deal with them during the data collection process. Additionally, practical aspects have been listed as recommendations for future studies involving this population.

The main limitation of this study was to have restricted the researchers’ records to field diaries and a form with open-ended questions. Verbal manifestations during the interviews could have revealed new or different perceptions from what was recorded. However, all material obtained was analyzed. The information on the forms at the end of the data collection period had similar content to that recorded during the process but was more synthesized and systematized. Thus, these were complementary methods that demonstrated consistency in the perceptions of the researchers’ experiences.

**Table 5:** Challenges and recommendations for planning a study involving older people in long-term care facilities

*Challenges faced during the survey process*	*Approach to overcome the challenges*
The complex clinical and functional profiles of older adults often resulted in refusal, resistance, and low cooperation during data collection. Moreover, rapid functional decline further complicated the research process, resulting in the loss of participants.	The researchers aimed to conclude data collection in the first and only attempt to approach older people whenever possible. However, when it was not possible to engage with the older adults initially, additional attempts were made to secure their involvement, considering emotional and health-related fluctuations.
A high prevalence of cognitive impairment among participants was observed, with low levels of cooperation and behavioral issues such as depression and irritability.	Researchers demonstrated empathy, patience, and flexibility to adapt to each individual’s unique needs and limitations, particularly those with cognitive impairments.
Communication with some older adults was complicated by speech difficulties, lack of interest, and sensory impairments such as hearing and vision loss.	Researchers sought to communicate directly and clearly with older adults by speaking louder and more slowly, using appropriate language, establishing non-verbal communication, and using gestures and facial expressions. Whenever possible, caregivers supported communication in more complex cases.
The listing of older adults in some LTCFs was outdated, containing individuals who have already passed away.	There was a need to continuously update the records of older adults within LTCFs before the sample selection.
Some LTCF coordinators have shown significant resistance and skepticism towards the research.	Flexibility was needed to justify the study’s importance and demonstrate potential benefits, such as enhanced resident care and scientific evidence generation.
Obtaining informed consent from LTCF coordinators and older residents, especially those with severe cognitive impairment, was a challenge.	Collaborative relationships with LTCFs were crucial for addressing concerns. We also implemented flexible recruitment strategies, such as repeated telephone calls, personal visits, and tailored explanations of the study’s importance. Careful consideration of consent by proxy and respecting the autonomy of individuals with cognitive impairment was necessary.
Institutional routines affected the execution of the study.	There was a need for adjustments in data collection timeframes to accommodate residents’ schedules and activities. Coordination with LTCF staff to find suitable interview times while minimizing routine disruption was essential.
Logistics related to data collection, such as mobility issues among residents, finding suitable and private examination locations, and addressing biosafety concerns during the COVID-19 pandemic, particularly challenged the oral examination process.	The oral examinations were performed in available spaces, such as TV armchairs or beds, ensuring participant comfort and privacy. Adaptation and adherence to strict protocols were necessary to ensure successful data collection while safeguarding the health of residents and researchers. Standardized procedures for data collection were established, with protocols for each stage of the research process, and researchers received training.
Variables associated with subjective aspects posed challenges in assessing older adults with cognitive impairment. Traditional instruments often failed to capture their experiences accurately, raising concerns about the reliability of data collected from individuals with dementia.	We could not collect subjective variables from all older adults, only among those without cognitive impairment. However, we acknowledged the necessity for more context-specific tools tailored to individuals with dementia. These tools should address their unique communication challenges and cognitive abilities, enabling more accurate subjective assessment. Adopting a mixed-methods approach that combines qualitative and quantitative methods can offer a more comprehensive understanding of older adults’ subjective experiences, including those with dementia.
*Recommendations for future studies*	
Align Expectations: Ensure clear communication and mutual understanding between the researcher and the LTCF, outlining the indirect benefits of the study to engage the institutional community effectively.	
Establish Trustful Communication: To build a trustworthy relationship, foster open and constant dialog with the LTCF coordinators, caregivers, nursing staff, and, most importantly, the residents.	
Flexibility and Patience: Be adaptable, patient, and creative in establishing satisfactory communication with participants, considering the diversity of institutional contexts.	
Tailored Research Methods: Employ combined research methods and techniques tailored to the clinical-functional profile of the residents. Utilize multiple sources or methods to assess the same variables, such as consulting records, conducting interviews with residents, or using a proxy informant.	
Inclusion of Cognitive Impaired Individuals: Do not exclude individuals with cognitive deficits; their inclusion is crucial for delineating the complete profile of the population.	
Time Planning: Plan the research timeframe thoughtfully, considering the time required for LTCF recruitment and respecting the institutional routines.	
Optimizing Data Collection: Optimize data collection time with residents to avoid fatigue and discomfort, ensuring a positive experience for participants.	
Privacy Considerations: Choose suitable locations within the LTCF where residents feel at ease during examinations, ensuring privacy and comfort.	
Respect Autonomy: Respect the autonomy of residents who may choose not to participate at a given time; be open to their willingness to participate on another occasion.	

## Conclusion

The researchers recognized the important role played by LTCF coordinators and formal caregivers, underscoring the significance of empathetic methodologies and streamlined data collection processes in mitigating the challenges inherent to research conducted within LTCFs. The institutional context significantly influences the planning and execution of research involving older adults residing in LTCFs, particularly those with clinical-functional profiles that necessitate specific tailored approaches. Respecting older adults’ autonomy and establishing effective and respectful communication are fundamental for building trust. Recognizing the caregivers’ knowledge provides valuable understanding for the data collection process. The LTCF willingness to participate in the research reflects their commitment to advancing knowledge in the field while upholding institutional routines and residents’ well-being. Beyond methodological considerations, such as selecting appropriate variables, defining the sample, and employing valid measures, social and cultural aspects of the LTCFs can impact costs, required human resources, and the execution timeline. In conclusion, conducting studies in LTCFs demands careful planning, effective communication, and flexibility to address institutional and residents’ diverse profiles. Collaborating closely with LTCF staff and caregivers is essential for successful data collection and ultimately benefiting this vulnerable population.

## Data Availability

The datasets used and/or analysed during the current study are available from the corresponding author on reasonable request.
